# Water-Mediated Ionic Migration in Memristive Nanowires with a Tunable Resistive Switching Mechanism

**DOI:** 10.1021/acsami.0c13020

**Published:** 2020-10-14

**Authors:** Gianluca Milano, Federico Raffone, Michael Luebben, Luca Boarino, Giancarlo Cicero, Ilia Valov, Carlo Ricciardi

**Affiliations:** † Department of Applied Science and Technology, Politecnico di Torino, C.so Duca degli Abruzzi 24, 10129 Torino, Italy; ‡ Advanced Materials Metrology and Life Science Division, INRiM (Istituto Nazionale di Ricerca Metrologica), Strada delle Cacce 91, 10135 Torino, Italy; § Institute for Materials in Electrical Engineering II, RWTH Aachen University, Sommerfeldstrasse 24, 52074 Aachen, Germany; ∥ JARA—Fundamentals for Future Information Technology, 52425 Jülich, Germany; ⊥ Peter-Grünberg-Institut (PGI 7), Forschungszentrum Jülich, Wilhelm-Johnen-Straße, 52425 Jülich, Germany

**Keywords:** nanowires, resistive switching, memristive devices, moisture, ionics

## Abstract

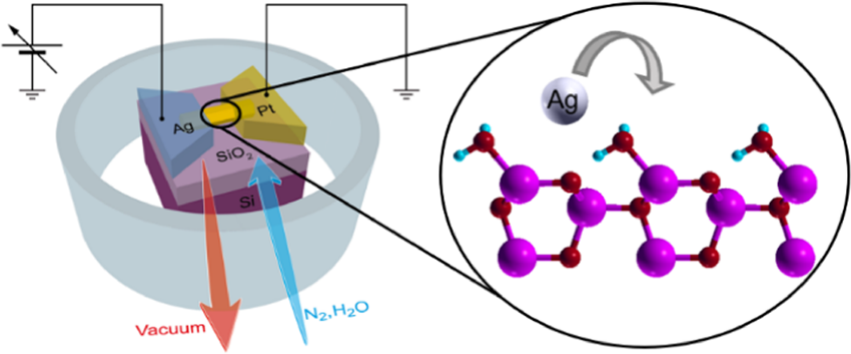

Memristive devices based on electrochemical resistive switching effects have been proposed as promising candidates for in-memory computing and for the realization of artificial neural networks. Despite great efforts toward understanding the nanoionic processes underlying resistive switching phenomena, comprehension of the effect of competing redox processes on device functionalities from the materials perspective still represents a challenge. In this work, we experimentally and theoretically investigate the concurring reactions of silver and moisture and their impact on the electronic properties of a single-crystalline ZnO nanowire (NW). A decrease in electronic conductivity due to surface adsorption of moisture is observed, whereas, at the same time, water molecules reduce the energy barrier for Ag^+^ ion migration on the NW surface, facilitating the conductive filament formation. By controlling the relative humidity, the ratio of intrinsic electronic conductivity and surface ionic conductivity can be tuned to modulate the device performance. The results achieved on a single-crystalline memristive model system shed new light on the dual nature of the mechanism of how moisture affects resistive switching behavior in memristive devices.

## Introduction

Memristive devices based on nanoionic redox processes are considered one of the most promising candidates not only for the realization of next-generation memories but also for the emulation of brain functionalities through the implementation of neuromorphic-type data processing.^1−7^ Despite recent breakthroughs in the implementation of neuromorphic algorithms in large memristive networks,^8−12^ detailed understanding of the complex redox behavior and of the ionic processes at the single-device level still represents a challenge. Redox-based memristors are two terminal devices in which the functionalities are enclosed in the resistive switching properties of a solid electrolyte, usually a metal-oxide film, sandwiched in between two metal electrodes.^13−17^ In the established literature, the switching mechanism is related to nanoionic processes of migration of oxygen species (valence change memory effect, VCM) or host metal ions from an electrochemically active electrode (electrochemical metallization memory effect, ECM), or both.^18^ Despite the great efforts in investigating the switching mechanism, the role of extrinsic effects such as the surrounding environment in the memristive behavior still needs to be elucidated. In particular, the influence of moisture on resistive switching has been raised as one of the most important open issues related to the comprehension and control of resistive switching events.^19^ Moisture incorporated from the surrounding environment or during device fabrication was reported to significantly influence the resistive switching properties of devices, representing concurring but also essential nanoionic processes for proper operation of memristive cells.^20−30^ However, no studies have considered the primary influence of adsorbed or absorbed moisture on the intrinsic electronic properties in memristive devices. Amorphous or polycrystalline films often have variable physicochemical properties such as structure, chemical composition, and stoichiometry, which can vary during switching operations. These materials can also dissolve and/or incorporate ions and/or atoms and even clusters during operations, and a different distribution of active places/centers can be expected. The density and nanoporosity of a given material can vary depending on the deposition conditions, resulting in a different ability to incorporate water molecules.^31^ Moreover, in metal-oxide thin films, ambient moisture as a parallel and concurring redox reaction and a source of different ionic species can suppress or enhance the coexistence of VCM and ECM mechanisms.^32^ From the technical point of view, the vertical device structure does not allow us to precisely define the high-quality interface between oxide and moisture where the processes could be clearly observed and studied. In this context, devices based on nanostructures with high surface-to-volume ratios have been proposed as an essential step for investigating the role of the surrounding environment in resistive switching phenomena.^33−35^ However, no suitable model system has been proposed to date.

In this work, we experimentally and theoretically investigate the interplay and dual influence of moisture on the resistive switching properties of a monocrystalline ZnO nanowire (NW) as a memristive model system. The individual NWs are brought into contact by means of asymmetric Ag and Pt electrodes to form an electrochemical metallization memory (ECM) cell with a planar structure.^4^ Both adsorption of water molecules from the surrounding environment as well as ionic migration are spatially restricted on the NW surface, making these devices an ideal (nano)platform for the investigation of the role of ambient in the physical mechanism of switching. Moisture absorbed on the NW surfaces modifies the NW band structure and decreases its electronic conductivity. In addition, adsorbed water molecules not only reduce the electroforming voltage but also facilitate subsequent switching cycles. According to *ab initio* density functional theory (DFT) calculations, this influence can be interpreted in terms of reduction of the energy barrier for ionic migration of Ag^+^ species on the crystalline NW surface in the presence of adsorbed water molecules. On the contrary, same devices in a dry environment show unreliable and stochastic switching characteristics or no forming at all.

## Experimental Methods

### NW Synthesis

ZnO NWs were realized by means of low-pressure chemical vapor deposition (LP-CVD) in a quartz horizontal tubular furnace, following the procedure previously reported.^36,37^ In brief, a Pt thin film used as a catalyst was placed in a quartz tube on an alumina boat surrounded by a Zn foil (purity 99.99%) that was used as a Zn source. The CVD process was performed at 650 °C for 20 min by flushing 300 sccm of Ar as a carrier gas and 200 sccm of O_2_ as a gas precursor at a pressure of 1.6 Torr. As a result of this growth process along the [0001] direction, a high-density array of vertically aligned and hexagonal-shaped ZnO NWs was obtained on the Pt substrate. A cross-sectional SEM image of as-grown ZnO NWs is reported in the Supporting Information S1.

### Device Fabrication

Single NW memristive devices were realized by means of a combination of optical and electron beam lithography (EBL), as previously reported.^4,38^ Initially, ZnO NWs were mechanically transferred from the growth substrate onto a SiO_2_ insulating substrate that was pre-patterned with a probe circuit realized by optical lithography and Cr/Au deposition. Contact geometries to bring single isolated nanostructures into contact with the probe circuit were realized by means of EBL (FEI Quanta 3D Microscope) and subsequent metal deposition (thickness of 80 nm). To realize asymmetric contact of NWs with Pt and Ag electrodes, two subsequent EBL processes were performed. During all of these fabrication steps, ZnO NWs were not exposed to aqueous solutions to avoid corrosion of the surface.^39^

### Atmosphere-Controlled Electrical Characterization

Atmosphere-controlled electrical characterizations were performed in a customized probe station.^23^ The probe station is equipped with a chamber in which the atmosphere can be modified in terms of the gas composition. Measurements were performed in air, a N_2_ environment (dry), and at different levels of moisturized N_2_. Measurements at different moisture levels were performed in a N_2_ environment by fluxing N_2_ through a cascade of gas wash bottles filled with deionized water. The relative humidity in the chamber was measured by means of an analog humidity sensor. Before atmosphere-controlled measurement, the chamber is evacuated down to ∼10^–5^ mbar and then refilled with the desired gas compositions. Electrical measurements were performed after stabilization of the ambient conditions using a Keithley 2636A or a Keithley 2636B. In all measurements, the Pt electrode of the memristive cell was grounded, while the Ag electrode was positively biased. During the electroforming process and subsequent resistive switching cycles, a compliance current of 10 μA was applied to prevent the full breakdown of the device. All electrical measurements were performed at room temperature.

### Density Functional Theory Method

The computational analyses were performed within the density functional theory framework using the QUANTUM ESPESSO package.^40^ The Perdew–Burke–Eenzerhof (PBE)^41^ formulation of the general gradient approximation was used to approximate the exchange–correlation potential, while the electron–ion interaction was described with ultrasoft pseudopotentials.^42^ A plane-wave basis set with a 28 Ry cutoff was employed to model the wave functions and a 280 Ry cutoff was used to model the density. The ZnO NW was reproduced simulating the exposed (11̅00) surface (which is the lateral surface of the NW) represented by 12 layers of ZnO slabs with a 2 × 2 surface supercell and periodic boundary conditions. The supercell included a 15 Å vacuum layer in the direction perpendicular to the surface to avoid spurious interactions among replicas. The Brillouin zone was sampled with a (3 × 3 × 1) Monkhorst–Pack *k*-point grid. The potential energy surface analysis (PES) was performed by placing a Ag adatom at 15 evenly spaced positions in the irreducible part of the (2 × 2) surface cell at an initial distance from the surface atoms of 2.5 Å. The Ag atoms were allowed to relax in the direction perpendicular to the surface (*z*) only until the forces were smaller than 26 meV/Å.

## Results and Discussion

### Influence of Moisture on Electronic Conduction

**Figure 1:**
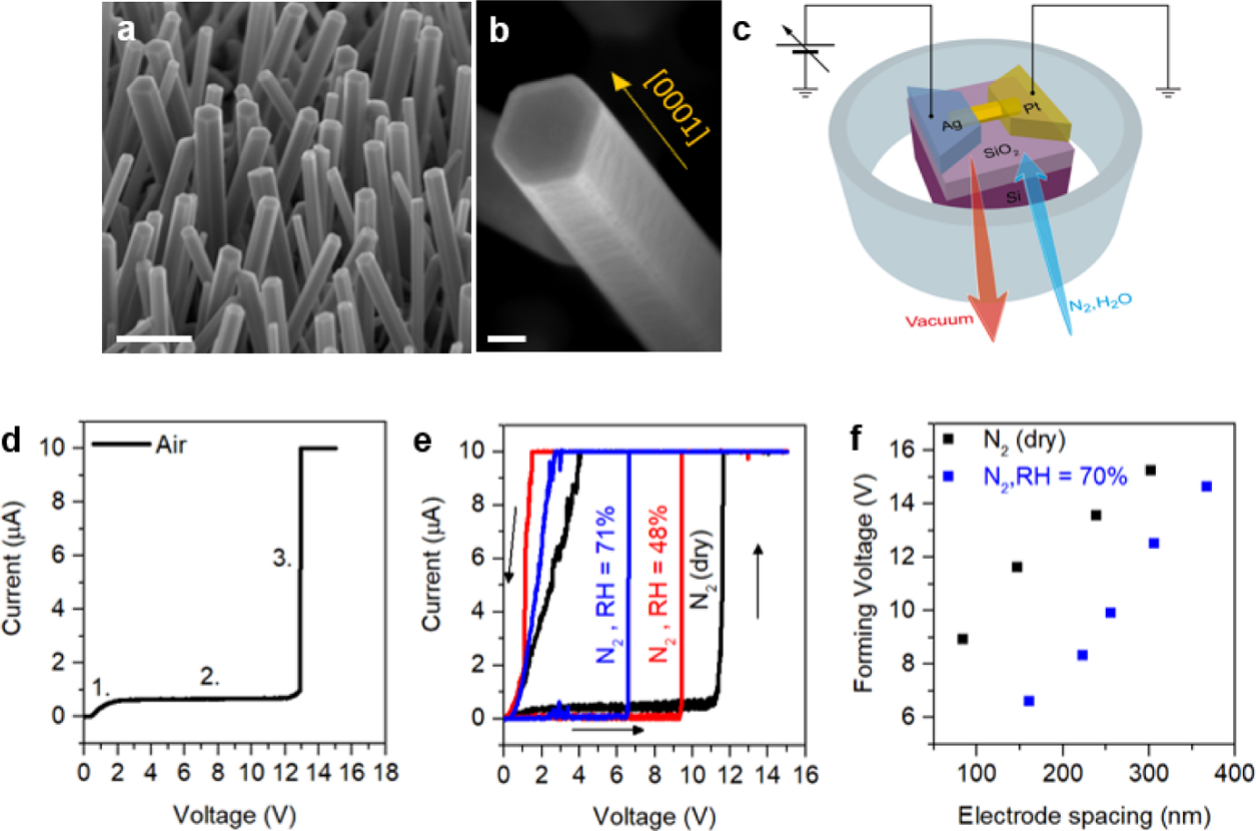
ZnO NW-based memristive device and influence of moisture on electroforming. (a) SEM image of NW arrays after the growth process (scale bar, 500 nm) and (b) details of a single ZnO NW with a hexagonal shape grown along the [0001] polar direction (scale bar, 40 nm). (c) Schematic representation of a single NW-based memristive device with electrical connections in an atmosphere-controlled chamber. (d) Typical electroforming process of a single NW memristive cell in air, evidencing a diodelike behavior for V <1.5 V (1.), current saturation regime for higher voltages (2.), and an abrupt change in resistance due to the formation of a Ag-conductive path (3.). (e) Comparison of the electroforming curves of NW memristive cells with the same electrode spacing (∼150 nm) in a N_2_ environment at different levels of moisture showing a reduction of the forming voltage in devices electroformed at higher levels of RH. (f) Electroforming voltage as a function of electrode spacing for different devices electroformed in a N_2_ environment under dry conditions and at RH = 70%, revealing a substantial reduction of the forming voltage in devices electroformed in the presence of moisture. Electrical measurements were performed at room temperature.

The synthesis of ZnO NWs was performed by LP-CVD (details in the Experimental Methods section), resulting in vertically aligned NWs as reported in Figure 1a. The median length and diameter of NWs are measured to be ∼1.6 μm and ∼100 nm (aspect ratio of ∼16) from the analysis of cross-sectional SEM images (Supporting Information S1). The hexagonal ZnO NWs have a wurtzite crystal structure with *P*6_3mc_ symmetry and the growth process proceeds along the [0001] polar direction,^37^ as shown in Figure 1b. A detailed structural and chemical characterization of ZnO NWs revealed that each NW is a monocrystal characterized by high chemical purity and a clean surface (no amorphous layers were observed on the NW surface), as investigated in our previous work.^37,39^ Single ZnO NWs dispersed on an insulating SiO_2_ substrate and brought into contact by means of asymmetrical Pt and Ag contacts were exploited as memristive model systems to investigate the influence of moisture on the resistive switching mechanism by means of atmosphere-controlled measurements, as schematized in Figure 1c. The device fabrication and experimental setup used for atmosphere-controlled measurements are discussed in the Experimental Methods section. It is worth noticing that, since the NW growth proceeds along the [0001] polar direction, surfaces exposed to the surrounding environment in single NW memristive devices are the nonpolar (11̅00).

Ag/ZnO NW/Pt devices can be considered as electrochemical metallization memory cells where device functionalities are regulated by ionic processes that are coupled to the electronic ones.^4,43^ In the pristine state, the electronic conduction mechanism is governed by Schottky barriers formed at the Pt/ZnO and Ag/ZnO interfaces and the device can be electrically described by means of two back-to-back connected Schottky diodes with the NW series resistance.^44^ In this context, *I–V* characterizations revealed that electronic conduction of the device is regulated by adsorbed species on the ZnO NW surface (Supporting Information S2). Among adsorbed species, water molecules play a key role in determining the formation of a depletion region with upward band bending on the NW surface, while no diffusion of water molecules in the crystalline ZnO bulk structure was reported.^45^ Depending on the moisture level, the amount of adsorbed water molecules on the NW surface regulates the extent of the depleted shell layer, resulting in a change of the effective diameter of the NW inner core that actively participates in the electronic conduction (a band diagram schematization is reported in Supporting Information S2). Therefore, an increase of the moisture level is responsible for an increase of the NW resistance. Also, our observations suggested that water electrolysis occurring when the device is biased can influence the electronic conduction mechanism of ZnO NWs, being responsible for an increase of resistance due to the creation of OH groups that can be consequently attached on the nonpolar lateral surfaces of the NW (details in Supporting Information S3).

### Influence of Moisture on Ionic Conduction

Despite influencing the electronic conduction mechanism, moisture can also strongly influence ionic conduction properties underlying the memristive behavior of single-crystalline ZnO NWs. In these devices, memristive functionalities are driven by the electrochemical metallization memory effect that involves dissolution of the electrochemically active electrode (Ag) and migration of metal ions along the NW surface under the action of the applied electric field to form a conductive path in between the electrodes.^4,46−49^ It is worth noticing that the coexistence of ECM and VCM mechanisms of switching can be safely excluded in these devices as a consequence of the high ZnO crystal quality.^4^ The initial assessment of the metallic path in between the electrodes can be performed by means of the so-called electroforming process, by applying a positive voltage sweep to the electrochemically active Ag electrode. The *I–V* characteristics of a typical electroforming process performed in ambient air are shown in Figure 1d. In the low-voltage range (1.), the device exhibited the characteristic of a forward-biased diode. On further increasing the voltage bias (2.), a current saturation regime was observed. This results from the Pt/ZnO junction that appears as an ohmic contact in the low-voltage range but acts as a reversely polarized Schottky barrier for high applied voltages.^4^ Then, the device transforms from a high-resistance state (HRS) to a low-resistance state (LRS) according to the forming voltage, where an abrupt current jump can be observed due to the formation of a Ag-conductive path along the NW (3.). Our results revealed that the electroforming process is strongly influenced by the moisture level in the surrounding environment. In a first experiment, voltage sweeps were applied to a single NW memristive cell with an electrode spacing of 376 nm by progressively increasing the amount of moisture in a N_2_ environment. While the formation of a conductive path was not possible in the case of dry N_2_ and relative humidity (RH) of 47%, a current jump confirming successful electroforming was observed when the humidity content was increased to RH = 71% (Supporting Information S4). In a second experiment, three different NW memristive cells with the same electrode spacing (∼150 nm) were electroformed at different moisture levels. The results reported in Figure 1e revealed that devices measured at higher moisture levels exhibited a severe reduction of the forming voltage. Indeed, the forming voltage decreased from about 11.6 to 9.4 and 6.6 V passing from dry N_2_ to RH = 48% and RH = 71%, respectively. Finally, the forming voltage measured in dry N_2_ or at RH = 70% as a function of electrode spacing for different single NW devices is reported in Figure 1f. Besides an expected decrease of the forming voltage by decreasing the electrode spacing, a substantial reduction of the forming voltage was observed for devices electroformed at RH = 70%. All of these results unequivocally show that moisture strongly influences the electroforming process, evidencing a substantial reduction of the forming voltage with an increase in the moisture level in the surrounding environment. It is worth noticing also that all electroforming processes were performed by applying a voltage sweep rate of 0.7 V/s, since the voltage sweep rate applied to the NW device was observed to strongly influence the forming voltage, as discussed in our previous work.^4^ In summary, the electroforming voltage can be modified and engineered by (i) tuning the electric field in between electrodes by adjusting the electrode spacing, (ii) regulating the device kinetics by regulating the voltage sweep rate of stimulation, and (iii) by tuning the ionic conduction mechanism by adjusting the level of moisture as discussed in the following section.

### Influence of Moisture on Resistive Switching Behavior

**Figure 2:**
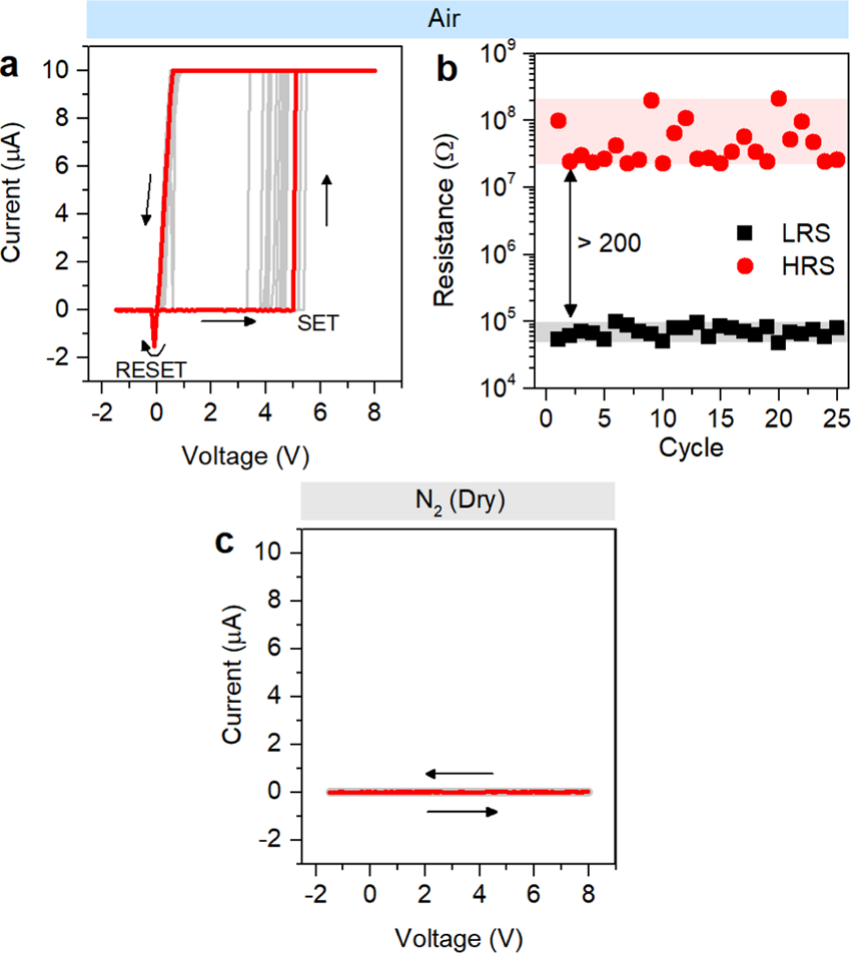
Influence of moisture on resistive switching behavior. (a) *I*–*V* characteristics showing bipolar resistive switching behavior of a single ZnO NW in air (RH ≈ 30%) and (b) corresponding HRS and LRS values over cycling extrapolated at a reading voltage of 0.4 V. (c) *I*–*V* characteristics of the same device in dry air (N_2_ environment) showing suppression of the resistive switching behavior. In both cases, the device was stimulated by means of 25 DC voltage sweeps in the voltage range −1.5/8 V. Electrical measurements were performed at room temperature.

Electroforming represents the ideal process for investigating the influence of moisture on the ionic transport mechanism since the device is initially in the pristine state and no metallic clusters are present on the NW surface. This means that ionic migration has to proceed along the entire electrode spacing before observing a change in the device resistance. However, it is shown here that subsequent resistive switching cycles, involving the formation/rupture of a metallic path due to the migration of Ag^+^ ions in between nanoclusters that are present on the NW surface after electroforming, are also affected by the moisture level. After electroforming, the device measured in air exhibited stable bipolar resistive switching characterized by an HRS/LRS ratio >200, as reported in Figure 2a,b. Endurance and retention characteristics of single ZnO NW-based memristive devices in air are reported in our previous work.^4^ After 25 cycles in air, the chamber was evacuated and then filled with N_2_. Under dry conditions, the device did not exhibit resistive switching since no SET events were recorded after device stimulation with repeated voltage sweeps as reported in Figure 2c. This means that resistive switching is suppressed in the absence of moisture, at least by considering the same range of applied voltage. In addition, suppression of resistive switching was directly observed in real time by reducing the moisture content in the surrounding environment over cycling (Supporting Information S5). It is worth noticing that the resistive switching behavior suppressed under dry conditions can be restored by exposing the device again to humidity (Supporting Information S6). All of these results reveal that ionic dynamics underlying resistive switching behavior are strongly influenced by moisture.

### Morphological Changes of the Device after Switching Events

**Figure 3:**
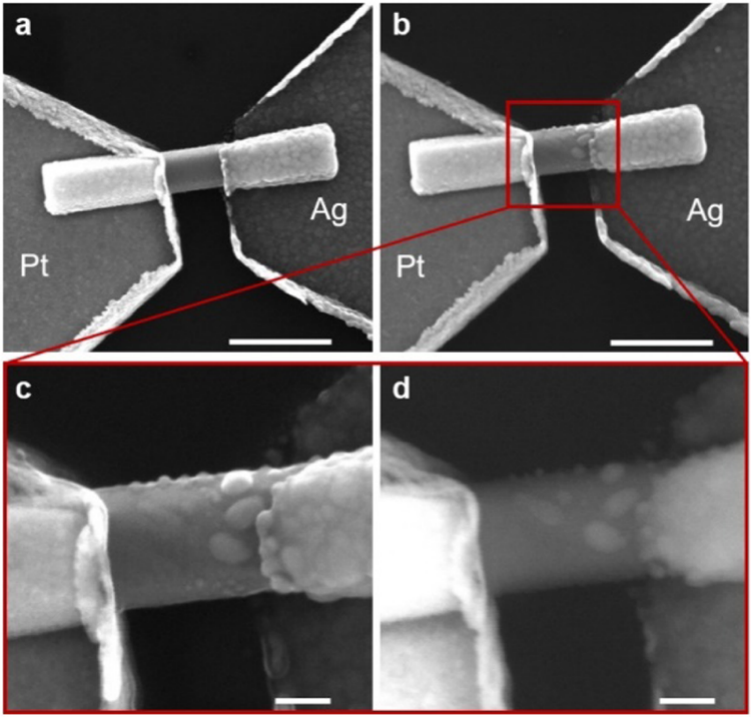
Change of device morphology after resistive switching. Scanning electron microscopy (SEM) image of (a) a single ZnO NW memristive device in the pristine state and (b) the same device after resistive switching (scale bars, 500 nm). Enlarged view of the device after resistive switching acquired with (c) secondary electrons and (d) backscattered electrons showing the presence of Ag nanoclusters on the ZnO crystalline surface accumulated mainly near the electrochemically active Ag electrode as a consequence of the low cation mobility (scale bars, 100 nm).

Due to the planar structure of the single NW memristive device and the restriction of ionic migration on the crystalline surface, ionic transport properties can be directly investigated by analyzing the morphology of the conductive path after resistive switching. While the pristine device exhibited a clean surface (Figure 3a), nanoclusters were observed on the ZnO crystalline surface after resistive switching events (Figure 3b). In particular, a high-resolution SEM image acquired with secondary electrons (SE) revealed details on the nanocluster size and distribution (Figure 3c), while imaging with backscattered electrons (BSE) was used to provide information on the nanocluster composition (Figure 3d). As BSE imaging is highly sensitive to differences in atomic number, making differentiation of metals based on contrast possible, it can be observed that the brilliance of nanoclusters is comparable to the brilliance of the Ag electrode, confirming that these nanoclusters are made of Ag. Note that these observations are in accordance with previous TEM and EDS analyses,^4^ corroborating the previously discussed switching mechanism. Interestingly, Ag nanoclusters were observed to be mainly accumulated near the Ag electrode, suggesting a conductive filament growth from the electrochemically active electrode toward the inert counter electrode. These filament growth dynamics are typical of memristive systems characterized by low cation mobility, where Ag^+^ ion transport represents a rate-limiting process for the conductive path formation.^50,51^ Moreover, it is important to point out that the interaction of water molecules with the ZnO surface can be responsible for corrosion of the crystalline oxide.^39,52^ However, while ZnO NWs exhibited a wrinkled and corroded surface after prolonged immersion in liquid water (Supporting Information S7), no significant corrosion effects were observed on exposing ZnO NWs to moisture and the smooth surface was preserved.

### Density Functional Theory (DFT) Simulations

**Figure 4:**
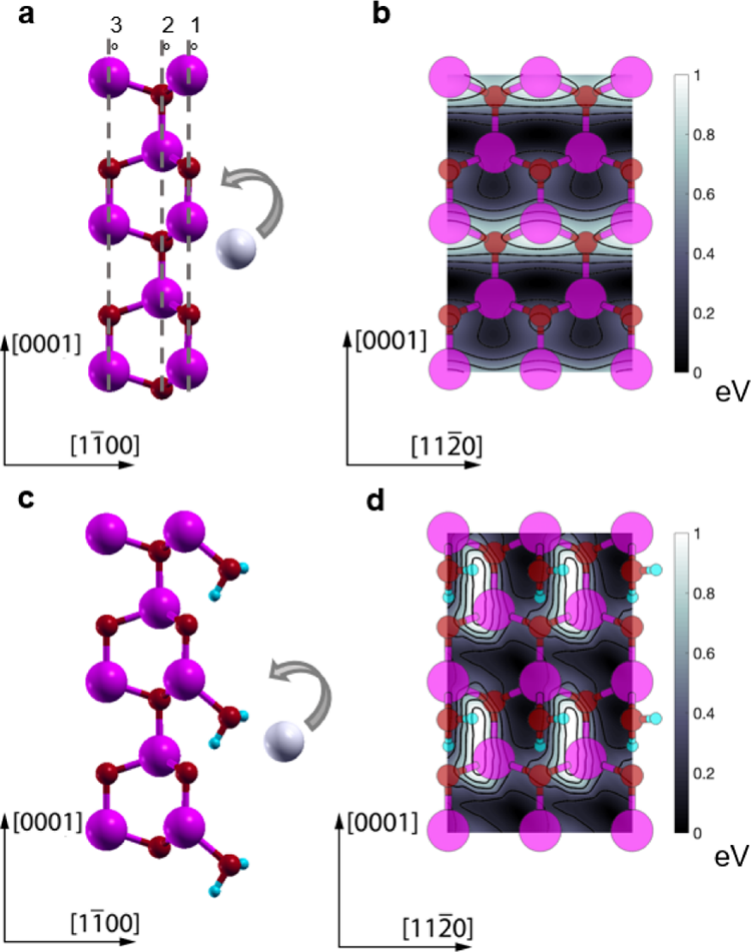
Theoretical investigation of the effect of adsorbed water on electric field-assisted Ag ion migration. Left side: ball and stick representation of a silver atom adsorbed on the ZnO NW bare (11̅00) surface (a) and on the ZnO NW (11̅00) surface with 1 ML of adsorbed water molecules (c) in a side view. Zinc, oxygen, hydrogen, and silver atoms are represented, respectively, in magenta, red, cyan, and gray. In panel (a), 1, 2, and 3° dashed lines indicate the positions of, respectively, the topmost, second highest, and third highest ZnO layers, respectively. Right side: top view and related potential energy surface for the adsorption of a silver atom on top of a bare ZnO (11̅00) surface (b) and on the water-covered ZnO (11̅00) surface (d) where the color bar was truncated to 1 eV and the zero energy was set to the lowest adsorption energy. Contour lines are plotted every 0.2 eV step.

In the following, we provide atomistic characterization of the drift process of Ag atoms on top of ZnO crystalline NW by means of density functional theory (DFT) simulations with the objective of understanding whether the presence of moisture molecules on the NW surface affects the rate of diffusion of silver. The details of the simulations are provided in the Methods section. We simulated the crystalline nanowire surface under two conditions: bare (Figure 4a,b) and with one monolayer (ML) of water (Figure 4c,d) adsorbed, representative, respectively, of the dry and high humidity conditions. High water pressure is, indeed, likely to cause the water molecules to adsorb at the NW surface. According to our calculations, the molecules attach to the Zn atoms belonging to the topmost surface layer (see Figure 4c,d), in agreement with previous studies on ZnO surfaces.^53^ One of the two hydrogen atoms of water forms a hydrogen bond with the NW oxygen surface atom, while the other is H-bonded to a nearby H_2_O molecule. Intermediate humidity stages will likely be characterized by bare and covered patches of different sizes. The different degrees of humidity will determine in general the amount of adsorbed water on the surface.

**Figure 5:**
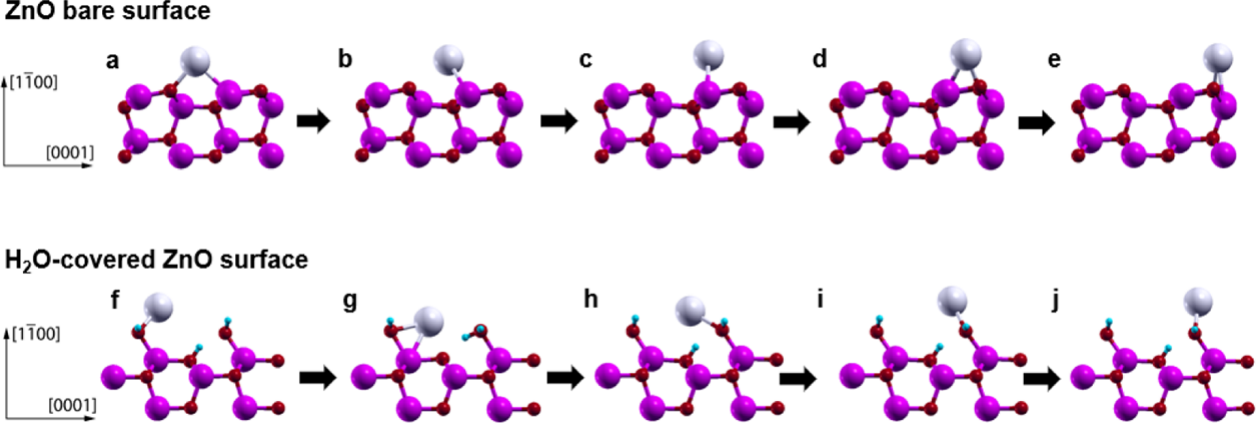
Ball and stick representations of the diffusion mechanism of a Ag atom at the bare (a–e) and H_2_O-covered (f–j) ZnO (11̅00) surfaces in the [0001] direction (side view). Zinc, oxygen, hydrogen, and silver atoms are represented, respectively, in magenta, red, cyan, and gray. When moving on the H_2_O-covered surface (f–j), the silver atom is always attached to a water molecule. This Ag–H_2_O bond has the double effect of flattening the PES and preventing the repulsive interaction between Ag and the surface Zn atoms.

For both bare and water-covered surfaces, we performed a PES for the adsorption of a single silver atom on the NW (11̅00) nonpolar lateral surfaces. The PES reveals the presence of diffusion barriers for a Ag atom along the NW axes and allows quantifying how likely the diffusion process is to occur as previous studies have shown.^49,54^ In the bare surface case, an analysis of Figure 4b shows that for the diffusion of Ag atoms in the growth direction of the NW [0001], two barriers must be overcome: a smaller one (0.03–0.17 eV depending on the direction of motion) and a larger one (0.64–0.78 eV). Notably, the largest one is located near the topmost Zn surface atoms, which interacts repulsively with Ag, both being positively charged metal species. The barrier represents the rate-limiting step of the drift process in vacuum. A representation of the diffusion path along the [0001] direction on the bare surface is shown in Figure 5a–e, where it is possible to see the Ag atom moving on top of the ZnO surface. From the most stable position (Figure 5a) located within a ZnO surface ring, the silver atom moves toward the uppermost Zn row (b–c), which, as mentioned, constitutes the primary obstacle for the diffusion. In the following steps (d–e), the adatom moves on top of the uppermost lying Zn–O dimer to reach the next ZnO ring. In these positions, the energy is lowered by the interaction with the surface oxygen atom. Concerning the perpendicular [112̅0] direction, there is no barrier preventing the lateral motion of silver atoms.

When the surface is covered by water, the diffusion mechanism changes drastically as indicated by the altered PES. Unlike the bare case, where the barriers are quite flat in the [112̅0] direction, the PES profile is highly uneven as a consequence of the orientation of the adsorbed H_2_O molecules. As can be seen in Figure 4d, when the Ag atoms are on the left side of a water molecule, the PES is particularly flat. This result can be understood by looking at the Ag as it moves in the [0001] direction (in Figure 5f–j). The metal atom almost never closely interacts directly with the surface, but rather travels on top of the adsorbed water molecules. A bond is formed between the silver atom and water molecules, which, in turn, split into OH^–^ and H^+^. A Ag–OH complex, bound to the surface, is formed, while the remaining H^+^ attaches to one of the oxygen atoms of the ZnO NW surface (compare Figure 5g,h). Because of the presence of the water molecules, when drifting in the electric field direction ([0001]), the silver atom avoids the repulsive interaction with the outermost Zn layer (Figure 5i) and, thus, the energy profile is rather flat as can be seen in Figure 4d. The limiting barrier is only 0.33 eV; the highest energy configuration corresponds to a highly stretched Ag–OH complex (see Figure 5g). If, instead, the silver atom moves on the right side of the adsorbed water molecules, the complex cannot be formed, so the PES presents much higher energy maxima.

The moisture has, therefore, a crucial effect on the switching process of ReRAM devices, enabling their operations thanks to the aiding effect of the water molecules covering the NW surface. A drastic change in the diffusion mechanism is observed. In the absence of moisture, surface Zn atoms suppress the movement of the adsorbed Ag atoms, hampering the ionic dynamics responsible for resistive switching. In contrast, when the humidity, and thus the surface coverage with water molecules, is high enough to ensure a percolation path where the Ag atoms can readily travel on top of the adsorbed water molecules avoiding the repulsive Ag–Zn interaction, the device is easily formed, as the diffusion barrier will be significantly lowered by the presence of water molecules.

Moreover, it should be pointed out that the water-mediated ionic migration mechanism with enhanced diffusion of Ag^+^ in a humid medium proposed here holds also in the presence of dissociated water or hydroxyl groups on the ZnO surface resulting from water electrolysis. Indeed, the major effect of the adsorbed water is to avoid the repulsive interaction between the moving Ag atom and the topmost Zn atom. This is achieved by dissociation of water into OH^•^ + H^•^ (where ^•^ indicates a bond with the surface). The OH^•^ dangling bond is saturated by the Ag atom moving in the direction of the electric field ([0001]). As the OH group is located exactly between the Ag and the topmost Zn, the repulsive interaction between them is negated. Because the single H^•^ adsorbed on the surface plays no role, we can assume that the same mechanism holds for a surface covered with only OH groups. Based on these results, electrolysis is suggested not to influence ionic conduction mechanism even if it is not possible to exclude that this effect can be involved in regulating the memristive cell electrochemistry participating in the counter electrode reaction as reported in thin-film-based devices,^19,21,23^ even if in our case we did not observe any bubbles or deformation of metallic electrodes related to electrolysis phenomena. Finally, it should be remarked that moisture is expected to support corrosion of the Ag-conductive path after its formation (i.e., in the ON state), leading to an expected poorer retention characteristic of the device.^19^

## Conclusions

In conclusion, we have experimentally and theoretically demonstrated the complex influence of moisture on ionic dynamics and electronic properties in a single-crystalline ZnO NW memristive model system. Adsorbed moisture decreases the electronic conductivity due to the modification of electronic surface properties, but decreases the forming voltage and ensures reliable device operation due to the influence on nanoionic processes underlying resistive switching effects. DFT calculations have shown that water molecules on the NW surfaces are responsible for a severe decrease of the diffusion barrier of Ag^+^ ions along the NW axis, facilitating the conductive filament formation. These results revealed the dual nature of adsorbed water molecules from ambient, playing a fundamental role in regulating the resistive switching mechanism.

## Supporting Information Available

As-grown ZnO NWs; effect of adsorbed species on electronic transport properties; influence of moisture on electroforming—additional data; direct observation of the effect of moisture on resistive switching; and reversibility of resistive switching behavior (PDF)
