# Supplementary material for: Water-Mediated Ionic Migration in Memristive Nanowires with a Tunable Resistive Switching Mechanism
Source: ACS Appl Mater Interfaces. 2020 Oct 14;12(43):48773–80. doi: 10.1021/acsami.0c13020 (PMC8014891; doi:10.1021/acsami.0c13020)
Supplement: Supplementary file 1 — am0c13020_si_001.pdf [file am0c13020_si_001.pdf]

# Water-mediated ionic migration in memristive nanowires with tunable resistive switching mechanism

*Gianluca Milano[1,2‡], Federico Raffone[1‡], Michael Luebben[3,4], Luca Boarino[2], Giancarlo Cicero[1], Ilia Valov[4,5 *], Carlo Ricciardi[1 *]*

[1]Department of Applied Science and Technology, Politecnico di Torino, C.so Duca degli Abruzzi 24, 10129 Torino, Italy.

[2]Advanced Materials Metrology and Life Science Division, INRiM (Istituto Nazionale di Ricerca Metrologica), Strada delle Cacce 91, 10135 Torino, Italy.

[3]Institute for Materials in Electrical Engineering II, RWTH Aachen University, Sommerfeldstrasse 24, 52074 Aachen, Germany.

[4]JARA – Fundamentals for Future Information Technology, 52425 Jülich, Germany.

[5]Peter-Grünberg-Institut (PGI 7), Forschungszentrum Jülich, Wilhelm-Johnen-Straße, 52425 Jülich, Germany.

Corresponding authors:

Ilia Valov: i.valov@fz-juelich.de;

Carlo Ricciardi: carlo.ricciardi@polito.it

‡G.M. and F.R. contributed equally to this work.

## S1: As-grown ZnO NWs

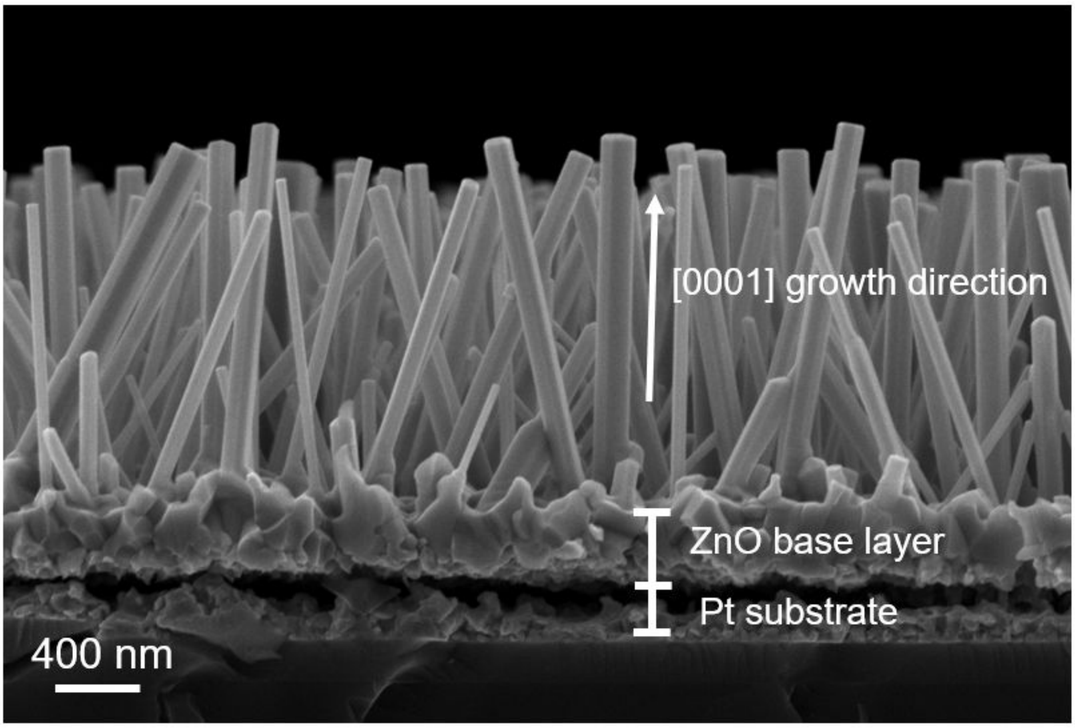


**Figure S1.** Cross-sectional SEM image of as-grown ZnO NW arrays. ZnO NWs grow along the [0001] direction while the polycrystalline ZnO base layer in between the NWs and the Pt substrate is resulting from the first stages of the growth process.

## S2: Effect of adsorbed species on electronic transport properties

A comparison of the *I-V* characteristic of the device pristine state in air and in vacuum is reported in Figure S2a, where the bias voltage was applied to the Ag electrode while the Pt electrode was grounded. Measurements were performed in a low voltage range in order to prevent ionic transport with consequent electroforming of the device. The asymmetric Ag/ZnO NW/Pt device exhibited a diode-like characteristic that can be understood in terms of formation of Schottky barriers at the metal-NW interfaces.[1,2] In particular, electronic conduction in the positive region is dominated by thermionic emission at the Ag/ZnO interface while the Pt/ZnO junction act as an Ohmic contact in the low voltage range.[2] The deviation of the *I-V* curves in the forward biased region, where the series resistance is becoming progressively more relevant in regulating the electronic transport, can be mainly attributed to a variation of the NW resistance. The increase of the NW resistance in air can be explained in terms of a different amounts of adsorbed molecules on the surface that act as charge traps. Among adsorbed species, water molecules acting as charge traps are responsible for upward band bending on the NW surface with the formation of a depleted shell layer free of electrons (Figure S2b), as theoretically and experimentally investigated in previous works[3,4]. The amount of adsorbed water molecules on the NW surface determines the extent of the depletion layer thickness and, thus, of the effective radius $r_{eff}$ that actively participate in electronic conduction. A schematization of the effect of adsorbed water species on ZnO band structure is reported in Figure 1b, where the fermi level $E_F$ is reported just below the conduction band due to the intrinsic n-type semiconductive behavior of ZnO arising from intrinsic donor-like defects.

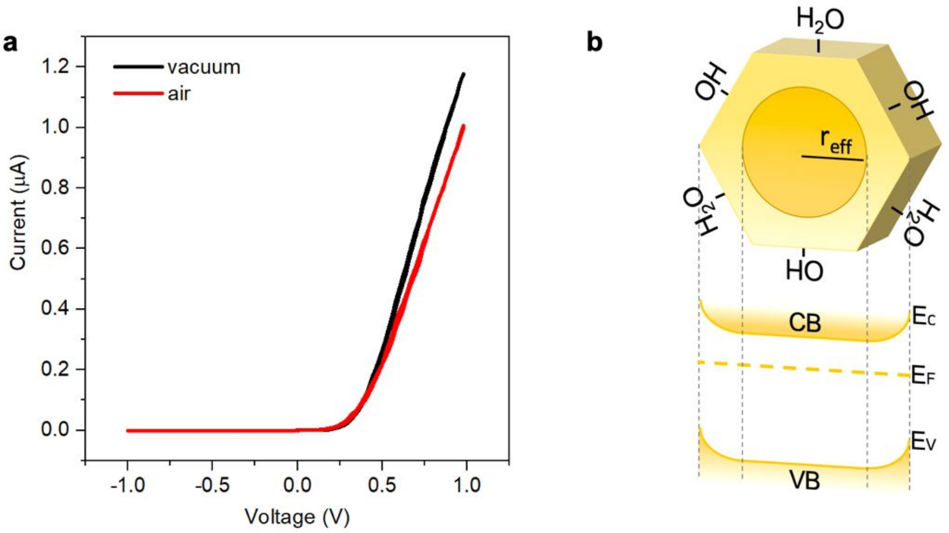


**Figure S2.** Effect of surroundings on electronic conduction of single ZnO NWs. a) Comparison of the *I-V* characteristic of the pristine state of a single NW memristive cell in air and in vacuum. b) Schematization of the effect of water-related species adsorbed on the NW on ZnO band structure. Water-related species results in upward band bending on the surface with consequent decreasing of the effective electronic conduction radius.

## S3: Influence of water electrolysis on electronic conduction

In order to investigate the influence of water electrolysis on electronic conduction, single ZnO NWs were contacted by means of electrochemically inert Pt symmetric electrodes and measured in atmosphere-controlled environment, as schematized in Figure S3a. In this fashion, it was possible to investigate the effect of water electrolysis on electrical conductivity without inducing ionic effects related to the electromigration of $Ag^+$ ions as observed in memristive Ag/ZnO NW/Pt devices.

Figure S3b reports *I-V* characteristic of the Pt/ZnO NW/Pt device measured in $N_2$ environment with a moisture level of RH=76%, showing a back-to-back diode like behavior arising from Schottky barriers at the Pt/ZnO interfaces[5]. Interestingly, as can be observed a continuous cycling of the device by means of *I-V* voltage sweeps resulted in a gradual decrease of the total conductivity of the NW. The decrease of the NW total conductivity can be interpreted as a direct consequence of electrolysis processes driven by the applied voltage difference, since the creation of OH groups that can be consequently attached on the nonpolar lateral surfaces of the NW is responsible for upward band bending occurring at the crystal surface with the creation of a depletion region free of electrons decreasing the overall NW conductivity and altering the Schottky Pt/ZnO interface, as theoretically analyzed in a previous works.[3] Also, it is necessary to point out that ionic conductivity related to the migration of $OH^-$ and/or $H^+$ species following the electrolysis process is negligible, since in this case an increase of the total conductivity of the device should be expected.

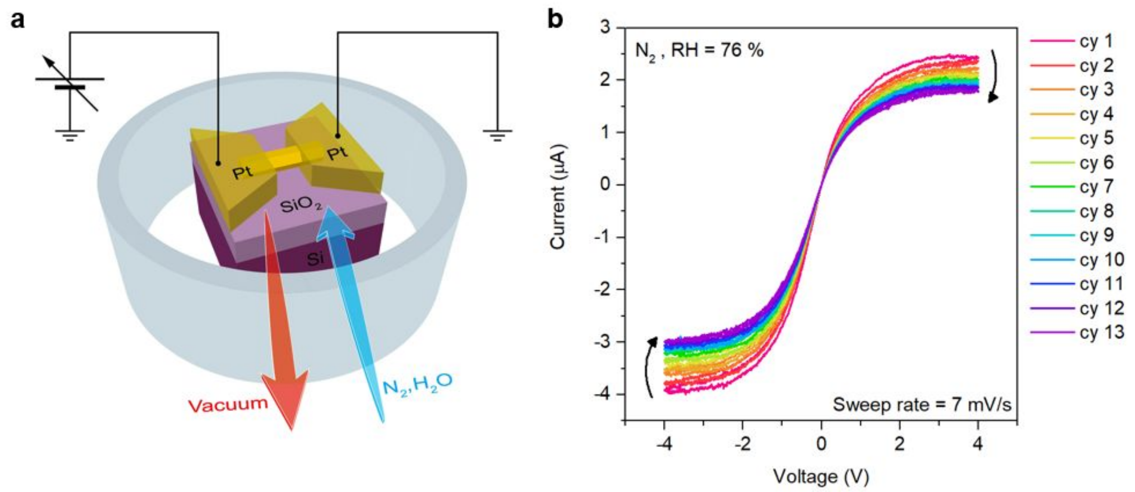


**Figure S3.** Influence of water electrolysis on electronic conduction. a) Schematic representation of a single ZnO NW contacted by means of Pt symmetric electrodes and electrically measured in a controlled atmosphere. b) *I-V* response of the device obtained by continuous *I-V* sweeping (sweep rate of 7 mV/s) in an $N_2$ atmosphere with RH = 76%.

## S4: Influence of moisture on electroforming – additional data

The influence of moisture on electroforming of ZnO NW memristive devices can be observed also by considering a device with high electrode spacing that cannot be formed in dry conditions. For example, even if a device with electrode spacing of 376 nm can be neither formed in dry conditions and at RH=47%, forming can be observed by increasing the moisture content to RH=71% as shown in Figure S3.

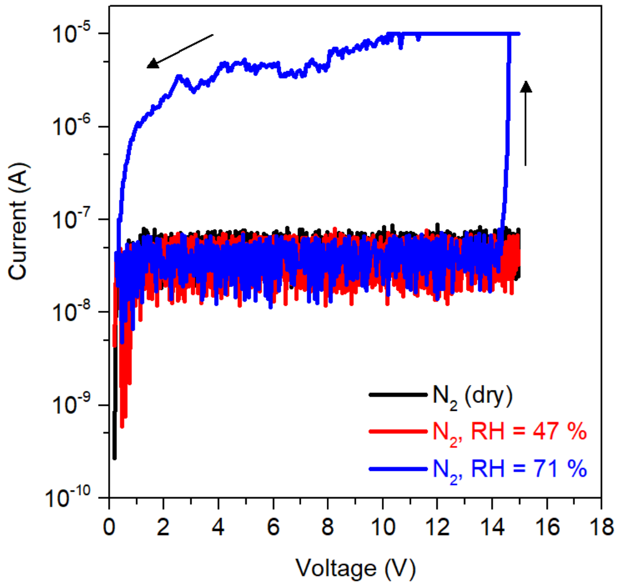


**Figure S4.** Electroforming process of a device with electrode spacing of 376 nm by progressively increasing the RH level in an $N_2$ environment (voltage sweep rate of 0.7 V/s). A successful electroforming was observed only when the moisture level was increased up to RH = 71%.

## S5: Direct observation of the effect of moisture on resistive switching

The effect of moisture on resistive switching can be further elucidated by changing the moisture content in the measurement chamber during cycling, as reported in Figure S4. Initially, the device was measured in air (RH ≈ 30 %) exhibiting bipolar resistive switching (see Cycle 1). After 25 cycles, a vacuum pump was started in order to evacuate the chamber and a humidity sensor was used to measure the progressive reduction of the moisture content. In the meantime, the electrical response of the device was continuously recorded. As can be observed from Figure S4, a progressive decrease of the moisture content resulted in a degradation of resistive switching performances with unstable SET processes (see Cycle 30) and subsequent total suppression of the SET events (see Cycle 40).

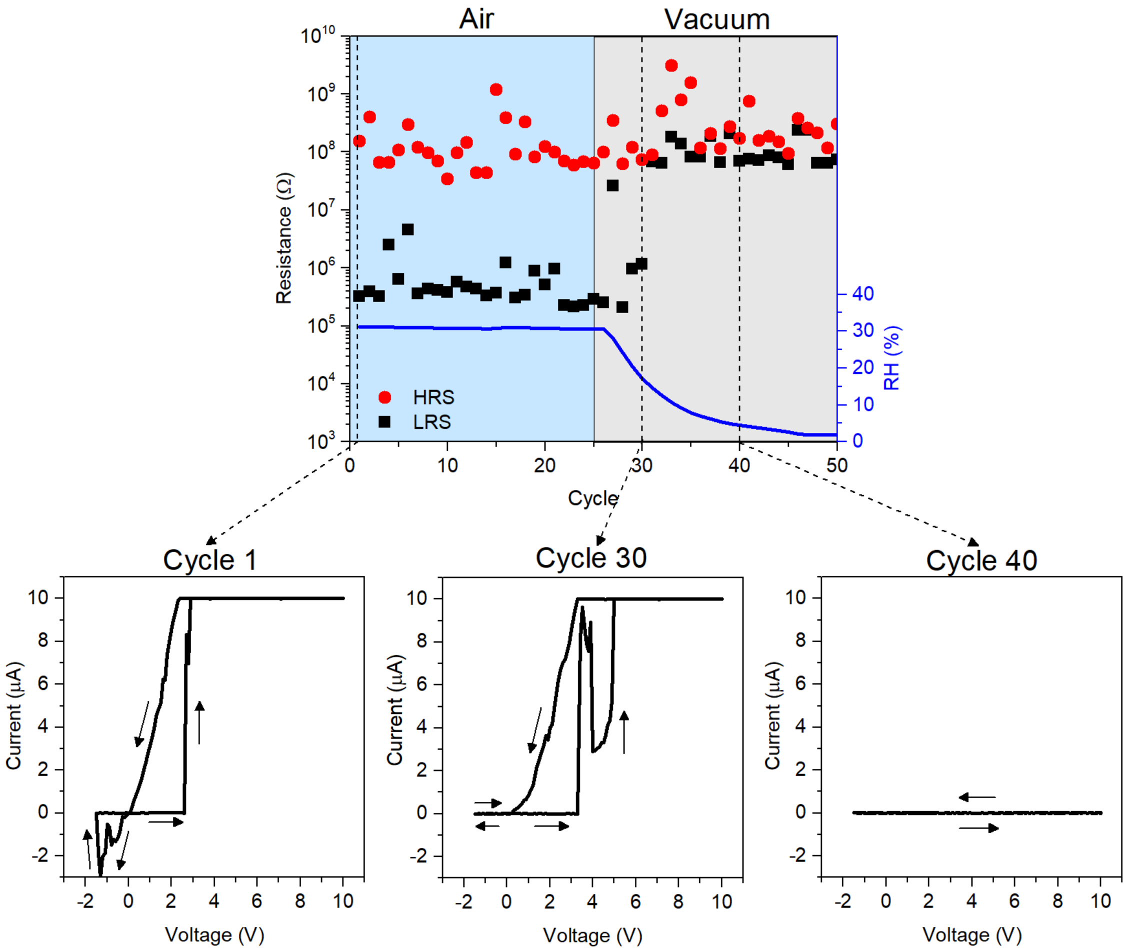


**Figure S5.** Direct observation of the effect of moisture on resistive switching behaviour. Dependence of the resistance states over cycling of a single ZnO NW memristive model system on RH. While the device exhibited resistive switching behaviour characterized by the presence of two resistance states in air, a progressive evacuation of the chamber with consequent reduction of the moisture content resulted in a gradual suppression of resistive switching behaviour with overlapping of LRS and HRS. Full *I-V* characteristics of significant cycles are reported. Note that evacuation of the chamber and desorption of water species from the NW surface is not instantaneous, influencing the gradual suppression of resistive switching.

## S6: Reversibility of resistive switching behavior

In order to check the reversibility of the resistive switching behavior after exposure to dry conditions, the device was cycled in air, then in vacuum and in air again. As reported in Figure S5, the device exhibited bipolar resistive switching in air (Figure S5 a), the resistive switching behavior was suppressed in vacuum (Figure S5 b) while resistive switching was subsequently observed to be restored again when the device was exposed to air again. Few cycles of stabilization were necessary before that the device exhibited again stable resistive switching passing from vacuum to air conditions.

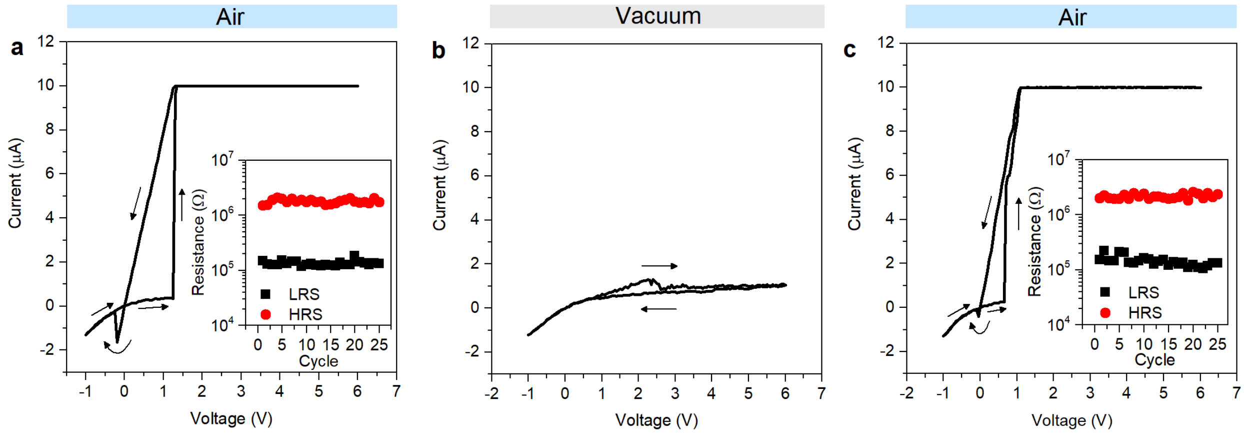


**Figure S6.** Reversibility of resistive switching behaviour. a) Resistive switching behaviour of a single ZnO NW memristor (HRS and LRS of 25 cycles as inset). b) After 25 cycles in air, the chamber was evacuated and the device was measured in vacuum, exhibiting suppression of resistive switching. c) The resistive switching behaviour can be restored by exposing the device again to air (HRS and LRS of 25 cycles as inset).

## S7: Interaction of ZnO NWs with water molecules and corrosion.

Indeed, the interaction of ZnO with water molecules can cause dissolution and corrosion according to the reaction[6–8]:

$$ZnO(s) + H_2O \rightarrow Zn^{2+} + 2OH^-$$

In order to observe experimentally the influence of water on the oxide corrosion, we have immersed ZnO NWs dispersed on a $SiO_2$ substrate in deionized water (pH ≈ 6.7−6.9, conductivity ∼150 µS/cm). In particular, we observed that a prolonged immersion of ZnO NWs in liquid water results in corrosion of the NW surface. As an example, we report the morphology of a single NW after immersion in water for 1 hour in Figure R6 a and b. While the NW surface after the growth process was smooth, a wrinkled and corroded surface can be observed with consequent diameter reduction and mass loss after interaction with water. However, no relevant corrosion was observed by exposing NWs to moisture.

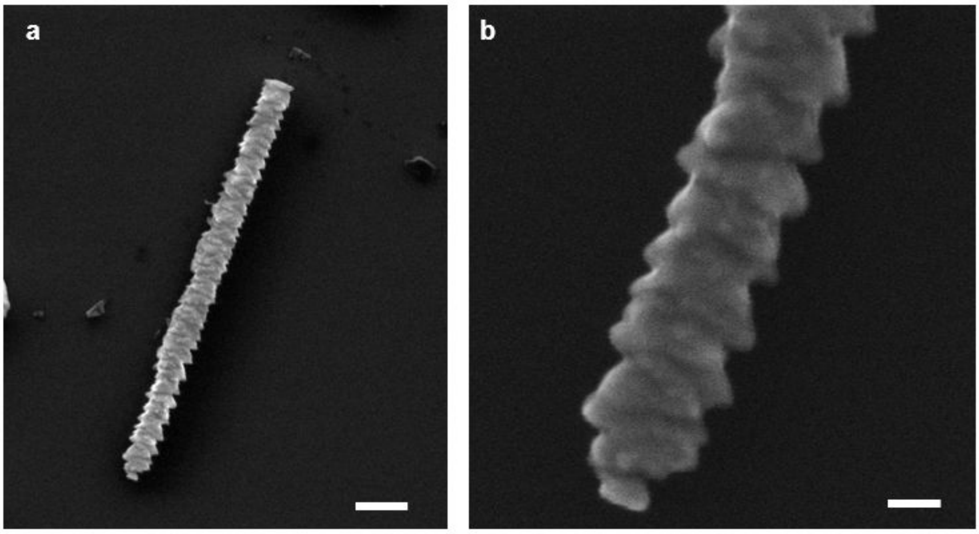


**Figure S7.** a) SEM characterization of a single ZnO NW deposited on $SiO_2$ substrate after being immersed in deionized water for 1 hour and b) detail of the NW where corrosion can be clearly observed.

## REFERENCES


(1) Milano, G.; Luebben, M.; Ma, Z.; Dunin-Borkowski, R.; Boarino, L.; Pirri, C. F.; Waser, R.; Ricciardi, C.; Valov, I. Self-Limited Single Nanowire Systems Combining All-in-One Memristive and Neuromorphic Functionalities. *Nat. Commun.* **2018**, *9* (1), 5151. https://doi.org/10.1038/s41467-018-07330-7.

(2) Milano, G.; Boarino, L.; Ricciardi, C. Junction Properties of Single ZnO Nanowires with Asymmetrical Pt and Cu Contacts. *Nanotechnology* **2019**, *30* (24), 244001. https://doi.org/10.1088/1361-6528/ab0a9c.

(3) Porro, S.; Risplendi, F.; Cicero, G.; Bejtka, K.; Milano, G.; Rivolo, P.; Jasmin, A.; Chiolerio, A.; Pirri, C. F.; Ricciardi, C. Multiple Resistive Switching in Core–Shell ZnO Nanowires Exhibiting Tunable Surface States. *J. Mater. Chem. C* **2017**, *5* (40), 10517–10523. https://doi.org/10.1039/C7TC02383A.

(4) Milano, G.; Luebben, M.; Laurenti, M.; Porro, S.; Bejtka, K.; Bianco, S.; Breuer, U.; Boarino, L.; Valov, I.; Ricciardi, C. Ionic Modulation of Electrical Conductivity of ZnO Due to Ambient Moisture. *Adv. Mater. Interfaces* **2019**, *6* (17), 1900803. https://doi.org/10.1002/admi.201900803.

(5) Miranda, E.; Milano, G.; Ricciardi, C. Compact Modeling of the I-V Characteristics of ZnO Nanowires Including Nonlinear Series Resistance Effects. *IEEE Trans. Nanotechnol.* **2020**, 1–1. https://doi.org/10.1109/TNANO.2020.2981214.

(6) Milano, G.; D'Ortenzi, L.; Bejtka, K.; Mandrile, L.; Giovannozzi, A. M.; Boarino, L.; Pirri, C. F.; Ricciardi, C.; Porro, S. Tuning ZnO Nanowire Dissolution by Electron Beam Modification of Surface Wetting Properties. *J. Phys. Chem. C* **2018**, *122* (14), 8011–8021. https://doi.org/10.1021/acs.jpcc.8b01158.

(7) David, C. A.; Galceran, J.; Rey-Castro, C.; Puy, J.; Companys, E.; Salvador, J.; Monné, J.; Wallace, R.; Vakourov, A. Dissolution Kinetics and Solubility of ZnO Nanoparticles Followed by AGNES. *J. Phys. Chem. C* **2012**, *116* (21), 11758–11767.

https://doi.org/10.1021/jp301671b.

(8) Jiang, C.; Hsu-Kim, H. Direct in Situ Measurement of Dissolved Zinc in the Presence of Zinc Oxide Nanoparticles Using Anodic Stripping Voltammetry. *Environ. Sci. Process. Impacts* **2014**, *16* (11), 2536–2544. https://doi.org/10.1039/C4EM00278D.
